# Clinical and laboratory findings of hookworms (*Ancylostoma* spp.) in naturally infected adult dogs

**DOI:** 10.29374/2527-2179.bjvm007724

**Published:** 2024-01-13

**Authors:** Bruna dos Santos, Naiara Mirelly Marinho da Silva, Silvia Eugenia Vargas Mora, André Augusto Justo, Elizabeth Moreira dos Santos Schmidt, Regina Kiomi Takahira

**Affiliations:** 1 Veterinarian, Programa de Pós-Graduação em Medicina Veterinária, Departamento de Clínica Veterinária, Faculdade de Medicina Veterinária e Zootecnia (FMVZ), Universidade Estadual Paulista “Júlio de Mesquita Filho” (UNESP), Botucatu, SP, Brazil.; 2 Veterinarian, Resident, Programa de Residência em Medicina Veterinária - Enfermidades Parasitárias dos Animais, Departamento de Clínica Veterinária, FMVZ, UNESP, Botucatu, SP, Brazil.; 3 Veterinarian. Pós-Graduação em Clínica Cirúrgica Veterinária, Departamento de Cirurgia, Universidade de São Paulo (USP), São Paulo, SP, Brazil.; 4 Veterinarian, DSc., Departamento de Clínica Veterinária, FMVZ, UNESP, Botucatu, São Paulo, Brazil.

**Keywords:** ancylostomiasis, canine, complete blood count, RDW, serum biochemistry, ancilostomíase, canino, hemograma, RDW, bioquímica sérica

## Abstract

Intestinal parasites of the genus *Ancylostoma* are the most prevalent in coproparasitological examinations and necropsies of dogs in Brazil. Although adult dogs often remain asymptomatic when infected, there is limited published information concerning the laboratory and clinical findings and severity of infection in symptomatic adult dogs. Therefore, this study aimed to characterize the clinical and laboratory findings of adult *Ancylostoma*-infected dogs. Data were obtained by surveying the medical records of dogs treated at a University Veterinary Hospital from June 2009 to June 2019. Of 243 cases, 30 met the selection criteria. The most frequent clinical signs were diarrhea and hematochezia (13/30 each - 43.3%), and the most common laboratory findings were eosinophilia (13/30 - 43.3%), increased RDW (6/29 - 20.7%), and hypoalbuminemia (5/21 - 23.8%). Dogs with *Ancylostoma* spp. presented an increased proportion of high RDW (P= 0.010) compared with non-parasitized dogs. Dogs with higher fecal egg scores (n= 18) showed significantly lower RBC (6.26 ± 0.64 *vs* 7.24 ± 0.74 10^6^/µL, P< 0.001) and albumin levels (2.8 ± 0.5 *vs* 3.2 ± 0.4 g/dL, P= 0.021), and an increased eosinophil count (1.42 ± 0.95 *vs* 0.58 ± 0.47 10^3^/µL, P= 0.003) compared with the control group. Fecal egg scores exhibited a weak correlation with eosinophils (r = 0.42, P= 0.002) and a moderate correlation with RBC (r = -0.52, P< 0.001). In conclusion, despite relevant clinical signs, the laboratory findings were indicative of mild alterations in adult dogs, especially in those with lower scores of eggs in coproparasitological tests.

## Resumo

Parasitas intestinais do gênero *Ancylostoma* são os mais prevalentes em exames coproparasitológicos e necrópsias de cães no Brasil. Os cães adultos frequentemente permanecem assintomáticos quando infectados. Entretanto, há pouca literatura sobre achados clinico-laboratoriais e gravidade da infecção em cães adultos sintomáticos. O objetivo do estudo foi caracterizar os achados clínicos e laboratoriais de cães adultos com *Ancylostoma* spp. Os dados foram obtidos de prontuários médicos de cães atendidos em um Hospital Veterinário Universitário de junho de 2009 a junho de 2019. Dos 243 casos, 30 atenderam aos critérios de seleção. Os sinais clínicos mais frequentes foram diarreia e hematoquezia (13/30 cada - 43,3%), além de eosinofilia (13/30 - 43,3%), aumento de RDW (6/29 - 20,7%) e hipoalbuminemia (5/21 - 23,8%). Os cães com *Ancylostoma* spp. apresentaram maior proporção de RDW elevado (P= 0,010) em comparação com cães hígidos; e aqueles com alto escore de ovos nas fezes (n= 18) apresentaram valores significativamente mais baixos de hemácias (6,26 ± 0,64 *vs* 7,24 ± 0,74 10^6^/µL, P< 0,001) e albumina (2,8 ± 0,5 *vs* 3,2 ± 0,4 g/dL, P= 0,021), bem como aumento da contagem de eosinófilos (1,42 ± 0,95 *vs* 0,58 ± 0,47 10^3^/µL, P= 0,003) em comparação com o grupo controle. Os escores de ovos nas fezes exibiram correlação fraca com eosinófilos (r= 0,42, P= 0,002) e moderada com hemácias (r= -0,52, P< 0,001). Concluiu-se que, apesar dos sinais clínicos relevantes, os achados laboratoriais indicaram alterações leves em cães adultos, especialmente naqueles com menores escores de ovos nos exames coproparasitológicos.

## Introduction

Intestinal parasites of the genus *Ancylostoma* are the most prevalent in coproparasitological tests and necropsies of dogs in Brazil (Dantas-Torres & Otranto, 2014; Oliveira-Arbex et al., 2017; Saldanha-Elias et al., 2019). Moreover, this genus represents the second most prevalent parasite in soil contamination of beaches, squares, sandboxes, and other recreational areas (Traversa et al., 2014). Both of the species that infect dogs in Brazil, *Ancylostoma caninum* and *Ancylostoma braziliense*, are zoonotic agents that can lead to eosinophilic enteritis and cutaneous larva migrans, respectively (Dantas-Torres & Otranto, 2014; Furtado et al., 2020); hence, they represent a significant public health concern.

In adult dogs, the prevalence of fecal samples positive for *Ancylostoma* spp. tends to be low (Nagamori et al., 2020), as does the elimination of eggs in feces when compared to dogs under one year of age (Santos et al., 2020). Nevertheless, animals older than one year can remain chronically infected due to the activation of hypobiotic larvae and reinfections (Gibbs, 1986). Although severe cases of blood spoliation and intestinal mucosal lesions have been reported in newborns and puppies, resulting in extreme anemia and hypoalbuminemia, infections in dogs over one year of age are often described as asymptomatic because of the resistance acquired with age (Bowman, 2014; Constantinoiu et al., 2015; Schmidt et al., 2016). Thus, there are a lack of data on clinical and laboratory findings in symptomatic conditions in adult dogs naturally infected with *Ancylostoma* spp.

The current retrospective study aimed to characterize the clinical and laboratory findings of adult dogs with hookworm parasitism treated at a Veterinary Teaching Hospital between 2009 and 2019. The hypothesis of the present study was that older dogs may demonstrate relevant laboratory alterations related to the *Ancylostoma* spp. infection, such as anemia, hypoalbuminemia, and leukocytosis, and that clinical signs may also be present.

## Materials and methods

### Study design

The medical records of the dogs admitted to the Veterinary Teaching Hospital at UNESP, Botucatu campus, from June 2009 to June 2019, were retrieved from an electronic database. All the data obtained from the medical records were accompanied by individual informed consent forms signed by the dogs’ owners. The information compiled was as follows: clinical history, physical examination, coproparasitological tests, and complete blood count (CBC). Serum biochemistry results were also collected, provided the tests were performed on the same day as the CBC. The data obtained from the adult dogs affected by *Ancylostoma* spp. were compared to reference intervals (RI), for CBC (Jain, 1993; Meyer & Harvey, 2004), serum biochemistry (Kaneko et al., 2008), and vital parameters (Feitosa, 2014).

### Inclusion and exclusion criteria

Dogs that tested positive for *Ancylostoma* spp. were retrospectively included in this study based on the fecal identification of eggs of parasites belonging to the family Ancylostomatidae, as determined by the simple flotation method. Moreover, dogs were also required to meet the following inclusion criteria: over 12 months of age or described as an adult, negative results for other intestinal parasites in coproparasitological tests using centrifuge-flotation and simple flotation methods, complete medical history (age, anamnesis, and physical examination), and an interval of less than or equal to seven days between the date of the CBC and the coproparasitological results. Dogs with concomitant systemic diseases reported in the medical records were excluded.

### Control group

To compare the laboratory findings between dogs affected and not affected by *Ancylostoma* spp., data on adult dogs from February to June 2019, healthy and free of intestinal parasites, were included. These animals were selected for the study because they were listed as donors of the Canine Blood Bank of the Veterinary Teaching Hospital at UNESP, Botucatu campus. All the animals weighed more than 20 kg, were between 1 and 8 years of age, and were not obese, as required for canine blood donors of this institution. In addition, the inclusion criteria for this study comprised: up-to-date polyvalent and anti-rabies vaccines, the absence of disease in the preceding two months, and a coproparasitological test with negative results by the simple flotation and centrifugal flotation methods. Animals with thrombocytopenia were excluded (platelet count below 160,000/µL) (Meyer & Harvey, 2004). All the dogs were tested for infectious diseases using the SNAP 4 Dx Plus test (IDEXX Laboratories, Maine, USA) (*Ehrlichia* sp., *Borrelia burgdorferi*, *Anaplasma* spp., and *Dirofilaria immitis*); two dogs tested positive for *Ehrlichia* spp., but were included in the study, following a negative PCR test.

### Clinical and laboratory evaluation

In addition to demographic data (sex, breed, age, city of origin) and clinical history, the following clinical variables were obtained: clinical signs, rectal temperature (RT, in Celsius), heart rate (HR, in beats per minute), capillary refill time (CRT, in seconds), degree of dehydration (not apparent, mild, moderate, or severe), and mucosal color (pink, pale, and congested).

The hematological variables evaluated comprised: red blood cell count (RBC), packed cell volume (PCV), hemoglobin, mean corpuscular volume (MCV), mean corpuscular hemoglobin concentration (MCHC), plasma protein, platelet count, and white blood cell count (WBC), as well as absolute counts of neutrophils, lymphocytes, eosinophils, basophils, and monocytes. The CBC was performed using the automated hematology analyzers Hema Screen 18 (Ebram Produtos Laboratoriales Ltda, SP, Brazil), from 2009 to 2014, and PocH-100iVDiff (Sysmex Corporation, Chou-ku, Kobe, Japan), from 2014 onwards. The morphological findings of erythrocytes and leukocytes, as well as the leukocyte differential, were described based on blood smears examined under an optical microscope (1,000x magnification). PCV was determined by microhematocrit capillary reading after centrifugation, and plasma protein by the refractometry method (Jain, 1993).

Urea, creatinine, alanine aminotransferase (ALT), alkaline phosphatase (ALP), gamma-glutamyl transferase (GGT), and serum protein, albumin, and globulin levels were measured using commercial kits (Labtest Diagnóstica, Lagoa Santa, MG, Brazil; Quibasa Química Básica Ltd., Santa Branca, MG, Brazil). The kinetic method was used for urea, ALT, GGT, and ALP measurements, and colorimetric assays for serum protein and albumin. The enzymatic method was applied to evaluate serum creatinine. Two automated analyzers were used: Cobas (Hoffmann La Roche, Basel, Schweizland) from 2009 to 2018, and Mindray Bs 200E (Shenzhen Mindray Bio-Medical Electronics Co., Ltd., Nanshan, Shenzhen, China) from 2018 onwards. Globulin levels were calculated by estimation, and their concentration was determined by subtracting albumin levels from serum protein levels (Stockham & Scott, 2011).

The methods used in the coproparasitological tests were simple flotation and centrifugal flotation in zinc sulfate (Faust et al., 1938; Willis, 1921). Additionally, a score was allocated according to the presence of eggs in feces (rare = absence of eggs in most microscope fields of view (FOV), 1+ = most FOV with eggs, 2+ = all FOV with 1 to 5 eggs, and 3+ = all fields of view with > 5 eggs), according to Santos et al. (2020).

### Data analysis

Statistical analysis was performed using GraphPad Version 8 software (GraphPad Software Inc., San Diego, CA, USA). The Shapiro-Wilk test and QQPlot were used to assess normality. Mean and standard deviation, along with median and minimum and maximum values, were used as measures of central tendency and dispersion. The one-way ANOVA and Kruskal-Wallis tests were used to compare parametric and non-parametric data, respectively. Tukey’s test was used for multiple comparisons of parametric data and Dunn’s test for non-parametric data. In the correlation analysis, the Pearson test was used for parametric variables and the Spearman test for non-parametric variables. Fisher’s exact test was used to compare proportions. Statistical significance was set at P < 0.05.

## Results

Of the 243 positive cases of *Ancylostoma* spp., 30 dogs (12.3%) met the selection criteria. The main reasons for exclusion were the presence of other endoparasites (n= 104), systemic disorders (n= 59), and missing information in medical records (n= 41). Two dogs presented the clinical disease twice in their history, with an interval of 17 and 24 months between the cases. Given the long period between infections, these were included in the study as independent cases.

The median age of the infected dogs was 36 (13 - 174) months, and five animals were described as adults, but without age definition. The sex prevalence was 46.7% (14/30) females and 53.3% (16/30) males. Among the dog breeds evaluated, 26.7% (8/30) were mixed breed and 73.3% (22/30) were purebred, i.e., Pitt Bull (7), Poodle (4), Labrador Retriever (2), German Shepherd dog (2), Cocker Spaniel (1), Dalmatian (1), Doberman (1), Golden Retriever (1), English Pointer (1) Rottweiler (1), and Yorkshire (1). Most of the animals lived in Botucatu (60.0%; 18/30) or in municipalities within a radius of 100 km from Botucatu (36.7% - 11/30): Avaré (4), Bauru (2), Bofete (2), Areiópolis (1), and Lençóis Paulista (1).

The selection of healthy dogs included 24 animals, 10 females (41.7%) and 14 males (58.3%), made up of Golden Retriever (4), Labrador Retriever (2), English Pointer (2), Chinese Shar Pei (2), one representative each of other purebreds (American Bully, Chow Chow, Great Dane, Brazilian Mastiff, German Shepherd, and Belgian Malinois), and six mixed-breed dogs. The median age was 36 (12 - 96) months, and the weight was 31.4 ± 9.7 kg. All the dogs lived in Botucatu, São Paulo, Brazil.

In the patient histories, only two cases did not report any clinical signs of infection. The most frequent signs were diarrhea (43.3% - 13/30) hematochezia (43.3% - 13/30), emesis (33.3% - 10/30), loose stool (23.3% - 7/30), and hyporexia (23.3% - 7/30). HR information was reported in 29 dogs, which presented values within the RI (60 to 160 bpm), with a mean ± standard deviation of 119 ± 22 bpm. Mild dehydration was reported in 10.7% (3/28), and in two dogs the mucosa was described as congested (3/30 - 10.0%). In addition, pale mucous membranes were present in three dogs (3/28 - 10.7%). Hyperthermia (RT > 39.2 °C) was reported in 20.7% of the cases (6/29). A CRT of three seconds was reported in four dogs (4/28 - 14.9%; RI = 1 to 2 seconds). In 26.7% (8/30), pain was reported in response to abdominal palpation. The score accolated for egg in feces were as follows: 20.0% (6/3) rare, 20.0% (6/30) 1+, 26.7% (8/30) 2+, and 33.3% (10/30) 3+. Table 1 presents the hematology and serum biochemistry data, compared with the RI. The main alterations observed in the hematology analyses were increases in RDW in 20.7% (6/29) and eosinophilia in 43.3% (13/30); and in the serum biochemistry analyses were increases in serum protein (5/22 - 22.5%) and hypoalbuminemia (5/21 - 23.8%).

**Table 1:** Hematological and serum biochemistry variables of adult dogs with *Ancylostoma* spp. infection compared with reference intervals, evaluated from 2009 to 2019.

**Parameters (Unit)**	**N**	**Mean ± SD**	**Median (Min-Max)**	**Reference interval**	**Dogs outside reference interval**	
					**Below % (N)**	**Above % (N)**
**Hematology values**								
RBC (10^6^/µL)	29	6.53 ± 0.73	6.50 (5.25 - 7.93)	5.50 - 8.50	6.9 (2)	0
Hemoglobin (g/dL)	30	15.2 ± 2.1	15.7 (11.3 - 20.7)	12 - 18	6.7 (2)	6.7 (2)
PCV (%)	30	44 ± 6	44 (33 - 59)	37 - 55	10.0 (3)	3.3 (1)
MCV (fL)	29	67.0 ± 5.3	67.7 (57.2 - 76.5)	60 - 77	6.7 (2)	0
MCHC (g/dL)	30	34.7 ± 2.3	34.7 (32.0 - 42.8)	32 - 36	0	13.3 (4)
RDW (%)	29	13.2 ± 1.9	13.2 (9.8 - 17.3)	Up to 15.0	**-**	**20.7** (6)
Plasma protein (g/dL)	30	6.8 ± 1.0	6.8 (4.2 - 8.2)	5.5 - 8.0	6.7 (2)	3.3 (1)
Platelets (10^3^/µL)	27	296 ± 120	267 (162 - 750)	160 - 430	0	7.4 (2)
WBC (10^3^/µL)	30	12.74 ± 4.38	12.05 (6.00 - 21.80)	6.00 - 17.00	0	**20.0** (6)
Neutrophils (10^3^/µL)	30	8.75 ± 3.67	8.15 (3.50 - 17.90)	3.00 - 11.50	0	**20.0** (6)
Lymphocytes (10^3^/µL)	30	1.76 ± 1.07	1.85 (0.30 - 4.20)	1.00 - 4.80	**20.0** (6)	0
Eosinophils (10^3^/µL)	30	1.56 ± 1.27	1.10 (0.10 - 6.00)	0.10 - 1.25	0	**43.3** (13)
Basophils (10^3^/µL)	30	0.05 ± 0.10	0 (0 - 0.4)	0 - 0.10	-	16.7 (5)
Monocytes (10^3^/µL)	30	0.63 ± 0.39	0.55 (0.10 - 1.60)	0.15 -1.35	0	6.7 (2)
**Serum biochemistry**								
Urea (mg/dL)	22	38.1 ± 13.2	38.0 (12.0 - 68.0)	21.0 - 59.9	4.5 (1)	4.5 (2)
Creatinine (mg/dL)	22	0.97 ± 0.21	1.00 (0.60 - 1.38)	0.50 - 1.50	0	0
ALT(U/L)	22	36.8 ± 25.1	31.5 (16.5 - 143.0)	21.0 - 102.0	4.5 (1)	4.5 (1)
ALP (U/L)	20	39.2 ± 28.2	32.0 (13.0 - 111.0)	20.0 - 156.0	**25.0** (5)	0
GGT (U/L)	21	3.1 ± 2.0	2.0 (0.6 - 7.6)	1.2 - 6.4	13.6 (3)	4.8 (1)
Serum protein (g/dL)	22	6.6 ± 0.9	6.7 (5.0 - 8.2)	5.1 - 7.1	4.5 (1)	**22.7** (5)
Albumin (g/dL)	21	2.9 ± 0.5	3.0 (2.0 - 3.7)	2.6 - 3.3	**23.8 (5)**	9.1 (2)
Globulin (g/dL)	21	3.7 ± 1.1	3.9 (1.8 - 6.1)	2.7 - 4.4	18.2 (4)	13.6 (3)

Reference Interval (Jain, 1993; Kaneko et al., 2008; Meyer & Harvey, 2004). Numbers in bold: more than 20% of the animals outside the reference interval. SD= standard deviation; Min-Max= minimum and maximum values; RBC= red blood cells; PCV= packed cell volume; MCV= mean corpuscular volume; MCHC= mean corpuscular hemoglobin concentration; RDW= red cell distribution width; WBC= white blood cells; ALT= alanine aminotransferase; ALP= alkaline phosphatase; GGT= gamma-glutamyl transferase.

According to fecal egg scores, the dogs were assigned to two groups: low score (rare and 1+, n= 12) and high score (2+ and 3+, n= 18). Clinical signs and aspects of the physical exam were compared using the Fisher’s exact test (Table 2), and pain in abdominal palpation was more observed in the low score group. The median (range) age in the low score group (36 [15 – 156]) was similar to that of the high score group (36 [13 – 174]). The laboratory variables of these two groups were also compared with each other and with the control group (Table 3). The animals in the high score group exhibited lower RBC, PCV, hemoglobin, and albumin, and higher WBC, neutrophils, eosinophils, monocytes, PTS, and globulins compared to of the dogs in the control group. The animals in the low score group presented higher platelet counts, WBC, eosinophils, and monocytes than those of the control group. None of the variables differed statistically between the low and high score groups. All the dogs presenting with anemia (PCV < 37%) belonged to the high score group. There was a significantly higher proportion of high RDW (P= 0.010) and basophilia (P= 0.024) in dogs with *Ancylostoma* spp. compared to healthy dogs.

**Table 2:** Percentage of clinical signs and findings of adult dogs with low score and high score of *Ancylostoma* spp. eggs, evaluated from 2009 to 2019.

**Parameters**	**Low**	**High**	**P-value**
	**egg score (%)**	**egg score (%)**	
High RT (>39.2°C)	18.2	22.2	>0.999
CRT 3 seconds	9.1	17.7	>0.999
Mild dehydration	8.3	12.5	>0.999
Pale or congested mucous	8.3	27.8	0.358
Pain in abdominal palpation	50.0	11.1	**0.034**
Emesis	33.3	33.3	>0.999
Diarrhea	41.7	44.4	>0.999
Hematochezia	41.7	44.4	>0.999
Hyporexia	25.0	22.2	>0.999
Mucus in stool	25.0	5.7	0.274
Loose stools	20.0	16.7	>0.999
Melena	16.7	22.2	>0.999
Apathy	16.7	16.7	>0.999
Anorexia	6.7	0	0.152
Weight loss	8.3	16.7	0.631
Polyphagia	8.3	11.1	>0.999

Fisher’s exact test was performed to compare groups. Numbers in bold: P <0.05. RT= rectal temperature; CRT= capillary refill time. Low egg score: n= 12; High egg score: n= 18.

**Table 3:** Comparison of hematological and serum biochemistry variables between adult dogs infected by *Ancylostoma* spp. presenting low egg score, high egg score, or no eggs in feces (control group), evaluated from 2009 to 2019.

**Parameters (Unit)**	**Low egg score group**			**High egg score group**			**Control group (n = 24)**		***P*-value**
	**n**	**Mean ± SD**	**Median (Min-Max)**	**n**	**Mean ± SD**	**Median (Min-Max)**	**Mean ± SD**	**Median (Min-Max)**	
**Hematology values**													
RBC (10^6^/µL)	12	6.86 ± 0.65	6.73 (5.84 - 7.93)	17	6.31 ± 0.64 ***	6.26 (5.25 - 7.86)	7.24 ± 0.74	7.32 (5.59 - 8.93)	**<0.001**
Hemoglobin (g/dL)	12	16.0 ± 2.0	15.8 (13.1 - 20.7)	18	14.7 ± 2.1**	14.1 (11.3 - 18.4)	17.0 ± 2.1	17.2 (13.1 - 21.2)	**0.003**
PCV (%)	12	46 ± 6	48 (37 - 59)	18	42 ± 6 **	44 (33 - 53)	49 ± 5	48 (39 - 57)	**0.004**
MCV (fL)	12	67.5 ± 5.3	66.8 (60.4 - 76.1)	17	66.5 ± 5.8	68.1 (57.2 - 76.5)	67.0 ± 3.6	68.7 (59.9- 71.6)	0.890
MCHC (g/dL)	12	34.7 ± 1.6	35.0 (32.2 - 37.9)	18	34.7 ± 2.7	33.9 (32.0 - 42.8)	35.1 ± 1.9	34.6 (33.0 - 41.5)	0.788
RDW (%)	12	12.7± 2.6	12.1 (9.8 - 17.3)	17	13.6 ± 1.3	13.5 (11.3 - 16.1)	12.5 ± 1.1	12.3 (11.0 - 14.8)	0.080
Plasma protein (g/dL)	12	6.5 ± 1.0	6.6 (4.2 - 8.2)	16	7.0 ± 1.0	6.8 (5.6 - 8.2)	6.5 ± 0.5	6.5 (5.6 - 7.4)	0.129
Platelets (10^3^/µL)	11	327 ± 167*	277 (168 - 750)	16	274 ± 71	264 (162- 410)	234 ± 57	234 (154 - 398)	**0.031**
WBC (10^3^/µL)	12	12.11 ± 4.59*	11.60 (6.00 - 20.80)	18	13.16 ± 4.32**	12.05 (7.60 - 21.80)	8.55 ± 1.99	8.50 (5.10 - 12.60)	**<0.001**
Neutrophils (10^3^/µL)	12	8.00 ± 3.17*	8.15 (4.00 - 13.70)	18	9.25 ± 3.97***	8.50 (3.50 - 17.90)	5.35 ± 1.39	5.22 (2.40 - 8.89)	**<0.001**
Lymphocytes (10^3^/µL)	12	1.66 ± 1.02	1.45 (0.30 - 3.70)	18	1.83 ± 1.13	1.95 (0.30 - 4.20)	2.25 ± 0.84	2.19 (0.66 - 4.03)	0.183
Eosinophils (10^3^/µL)	12	1.73 ± 1.68**	1.40 (0.10 - 6.00)	18	1.42 ± 0.95*	1.10 (0.30 - 3.40)	0.58 ± 0.47	0.44 (0 - 1.64)	**0.003**
Basophils (10^3^/µL) #	12	0.05 ± 0.10	0 (0 - 0.3)	18	0.05 ± 0.12	0 (0 - 0.4)	0 ± 0.02	0 (0 - 0.10)	0.177
Monocytes (10^3^/µL)	12	0.67 ± 0.38*	0.70 (0.10 - 1.50)	18	0.61 ± 0.41*	0.50 (0.10 - 1.60)	0.35 ± 0.23	0.33 (0 - 1.0)	**0.012**
**Serum biochemistry**													
Urea (mg/dL)	9	39.5 ± 12.2	43.0 (22 - 62.0)	13	37.1 ± 14.3	37.0 (12.0 - 68.0)	40.1 ± 13.0	36.5 (23.0 - 68.0)	0.800
Creatinine (mg/dL)	9	1.04 ± 0.19	1.06 (0.80 - 1.38)	13	0.92 ± 0.21	1.00 (0.60 - 1.30)	1.03 ± 0.15	1.04 (0.76 - 1.40)	0.118
ALT(U/L)	9	30.9 ± 6.7	30.0 (25.0 - 47.0)	13	40.9 ± 32.1	33.0 (16.5 - 143)	38.8 ± 7.3	39.0 (27 - 51)	0.423
ALP (U/L) #	8	39.8 ± 32.9	27.5 (14.0 - 111.0)	12	38.8 ± 26.1	32.0 (13.0 - 87)	37.5 ± 30.1	33.0 (13 - 172)	0.956
GGT (U/L)	8	3.8 ± 2.2	3.0 (1.0 - 7.6)	13	2.7 ± 1.9	2.0 (0.6 - 6.3)	3.3 ± 2.0	3.1 (0.1 - 8.7)	0.771
Serum protein (g/dL)	9	6.3 ± 0.8	6.2 (5.2 - 7.4)	11	6.8 ± 0.9*	7.0 (5.0 - 8.2)	6.1 ± 0.5	6.2 (5.2 - 7.7)	**0.017**
Albumin (g/dL)	8	3.0 ± 0.4	3.1 (2.3 - 3.6)	13	2.8 ± 0.5*	2.9 (2.0 - 3.7)	3.2 ± 0.4	3.3 (2.3 - 3.8)	**0.021**
Globulin (g/dL)	8	3.2 ± 1.0	3.0 (1.9 - 4.5)	13	4.0 ± 1.0***	4.0 (1.8 - 6.1)	2.9 ± 0.5	2.9 (1.9 - 4.0)	**<0.001**

Statistical difference in the comparison with control group:

* =P < 0.05.

** = P < 0.01.

*** = P < 0.001.

# Non-parametric data: Kruskal-Wallis Test followed by Dunn’s Test (multiple comparisons).

Parametric data: One-way ANOVA, followed by Tukey’s Test (multiple comparisons). Numbers in bold: P < 0.05. SD= standard deviation; Min-Max= minimum and maximum values; RBC= red blood cells; PCV= packed cell volume; MCV= mean corpuscular volume; MCHC= mean corpuscular hemoglobin concentration; RDW= red cell distribution width; WBC= white blood cells; ALT= alanine aminotransferase; ALP= alkaline phosphatase; GGT= gamma-glutamyl transferase.

Using data from both control and infected dogs, the Pearson test revealed a strong positive correlation between RBC and albumin levels (r= 0.70, P< 0.001). In the Spearman correlation test, the fecal egg score showed a moderate negative correlation with RBC (r = -0.52, P< 0.001) and a weak negative correlation with albumin (r = -0.38, P = 0.009). Additionally, the fecal egg score exhibited a weak positive correlation with eosinophil count (r = 0.42, P = 0.002).

## Discussion

The current study investigated the consequences of infection by *Ancylostoma* spp. in adult dogs, based on the clinical and laboratory findings. The target population of this study comprised dogs treated in a hospital setting; hence, it was easier to recruit the 30 selected dogs, since the demand for veterinary care increases when an animal shows signs of disease (Katagiri & Oliveira‐Sequeira, 2008).

Although important gastrointestinal clinical signs were observed, such as diarrhea, hematochezia, and emesis, which characterize acute ancylostomiasis, hookworm parasitism in adult dogs led to discrete consequences in the laboratory tests. Dogs with a high fecal egg score presented significant differences in several laboratory variables when compared to healthy dogs. Therefore, despite being symptomatic, milder systemic effects and hematological alterations may have been evidenced in older dogs due to the buildup of resistance with age, since the dogs were from an endemic area and may have been infected multiple times (Bowman, 2014; Constantinoiu et al., 2015).

The clinical signs described in the literature regarding experimental and natural infections in young dogs are similar to those observed in the dogs in the current study. The observed clinical signs, such as diarrhea, hematochezia, mucus in feces, emesis, melena, and apathy were likely triggered by intestinal mucosal laceration and hemorrhage (Bhanjadeo et al., 2023; Kopp et al., 2007). The mild dehydration and high CRT observed in some of the dogs are probably attributed to loss of fluid via the gastroenteric route due to the parasitism (Feitosa, 2014). Hyperthermia (> 39.2°C) and abdominal pain in response to palpation were also observed. Although the latter was more common in dogs with low fecal egg scores, but the cause of this difference is not clear. Interestingly, in human hookworm infections, the clinical gastrointestinal signs include vomiting, abdominal pain, hyporexia, hemorrhagic diarrhea or melena, and transient fever (Chapman et al., 2021; Neves, 2004; Wang et al., 2011), which are somewhat consistent to those documented in the current study.

The hematological alterations reported could be explained in terms of the blood loss, intestinal mucosal inflammation, and tissue larval migration related to the hookworm parasitism (Bowman, 2014; Constantinoiu et al., 2015; Stockham & Scott, 2011). In experimental settings, *Ancylostoma* spp. infections are characterized by anemia, leukocytosis by neutrophilia, lymphocytosis, and eosinophilia (Campos et al., 2018; Dias et al., 2013; Dracz et al., 2014). Basophilia is also frequently observed in parasitic diseases (Stockham & Scott, 2011). In general, the hematological alterations observed in this study were discrete in absolute numbers. The eosinophil count represented the variable with the highest proportion of dogs outside the RI, with 43.3% presenting eosinophilia. This cell type is directly related to the immune response against nematode parasites, which causes their numbers to increase in both the peripheral blood and the intestinal mucosa, even in animals with previous exposure to the parasite (Carroll & Grove, 1986; Dracz et al., 2014). In addition, eosinophilia is a relevant laboratory finding for the investigation of *Ancylostoma* spp. infection (Santos et al., 2020). Although hematological alterations occur physiologically due to aging, they generally remain within the RI for adult dogs. For instance, total protein and globulins increase, while hematocrit, MCV, serum iron, and albumin decrease with age (Radakovich et al., 2017). However, because the age of the dogs in the low and high score groups was similar, it is unlikely that the effect of age biased the present results.

The hematochezia and/or melena observed in 16 dogs provide evidence of the occurrence of blood spoliation associated with *Ancylostoma* spp. hematophagy, although the intensity of the loss was not high enough for the erythrogram variables to fall outside the RI in most of the dogs in the study, except for the RDW. This variable reads as the coefficient of variation of the RBC volume, and is deemed more accurate than the VCM for the detection of anisocytosis. In other words, increased RDW values imply the presence of small and/or large RBC (Miglio et al., 2023). Only three dogs presented anemia, which was mild and of the normocytic normochromic type, with two of these animals exhibiting an increased RDW. In addition, four non-anemic animals had elevated RDW values. In dogs with portosystemic shunts, a condition in which iron metabolism is altered due to hepatic dysfunction, RDW may be abnormally high even when anemia and microcytosis are absent. Furthermore, the RDW may fall within the RI in dogs with microcytosis (Martinez et al., 2019). Dogs with subclinical hookworm infection, but without anemia, have shown a decrease in iron levels (Schmidt et al., 2016). Although it is not possible to provide a definitive answer as to whether the observed anisocytosis arose from iron deficiency or a regenerative response, higher RDW coupled with normal MCV because of *Ancylostoma*-related iron deficiency emerges as a reasonable hypothesis.

The results of the biochemical tests applied to evaluate renal function and enzyme activity indicative of liver damage fell within the normal range according to the RI, or close to its limits. However, protein levels exhibited a different profile, with five dogs presenting with hypoalbuminemia and five with hyperproteinemia. In addition, dogs with a high fecal egg score showed lower albumin levels, and higher globulin and serum protein levels than the control group. These findings indicate an inflammatory component in this profile, associated with a possible enteric loss of albumin through exudation by mucosal lacerations and hemorrhage (Constantinoiu et al., 2015; Stockham & Scott, 2011), and potentially exacerbated by hyporexia. Even asymptomatic dogs parasitized by *Ancylostoma* spp. may present decreased serum albumin levels when compared to healthy dogs (Schmidt et al., 2016).

Significant correlations were observed of albumin, eosinophils, and RBC with the fecal egg score was observed. Statistical differences in laboratory variables according to the fecal egg score group, along with these findings, demonstrate that this value, although a semi-quantitative method, can indicate the intensity of intestinal parasitism. Campos et al. (2018) also found a moderate and negative correlation between albumin levels and the fecal egg count of *Ancylostoma* spp. (r= -0.57, P< 0.001). These correlations are possibly associated with the relationship between the fecal egg count and the number of parasites in the intestine (Carroll & Grove, 1984).

It should be noted that, as limiting factors, all the laboratory analyses and clinical evaluations were performed by different professionals and using different equipment over time, despite the standardization of laboratory techniques and clinical records. Accuracy errors inherent to the differential leukocyte count per 100 cells may have been reflected in the blood count. In addition, other diseases may not have been diagnosed when the dogs were treated at the University Veterinary Hospital.

Future investigations are necessary to elucidate the mechanism whereby some adult dogs, which are apparently immunocompetent, succumb to parasitism and become symptomatic, i.e., whether this is related to the intrinsic characteristics of the host or to peculiarities of the infecting species.

## Conclusions

In the adult dogs treated in the present Veterinary Hospital, *Ancylostoma* spp. infection caused some gastrointestinal signs, notably diarrhea and hematochezia. Hematological and biochemical alterations were generally mild, except for the elevated eosinophil count and RDW. Dogs with high fecal egg score showed significant decreases in RBC and albumin and increases in eosinophil counts compared to healthy dogs. The fecal egg score was negatively correlated with RBC and albumin levels and positively correlated with eosinophil counts. Thus, despite the marked clinical signs, the laboratory findings indicated a mild infection, particularly in dogs with lower fecal egg score.

## References

[B001] Bhanjadeo R., Patra R. C., Panda D., Sahoo R., Das D. P., Mohanty B. N. (2023). Comparative efficacy of ivermectin and fenbendazole against ancylostomiasis in dogs. Journal of Parasitic Diseases: Official Organ of the Indian Society for Parasitology.

[B002] Bowman D. D. (2014). Georgis’ Parasitology for Veterinarians.

[B003] Campos D. R., Perin L. R., Camatta N. C., Oliveira L. C., Siqueira D. F., Aptekmann K. P., Martins I. V. F. (2018). Canine hookworm: Correlation between hematological disorders and serum proteins with coproparasitological results. Revista Brasileira de Medicina Veterinária.

[B004] Carroll S. M., Grove D. I. (1984). Parasitological, hematologic, and immunologic responses in acute and chronic infections of dogs *with Ancylostoma ceylanicum*: a model of human hookworm infection. The Journal of Infectious Diseases.

[B005] Carroll S. M., Grove D. I. (1986). Response of dogs to challenge with *Ancylostoma ceylanicum* during the tenure of a primary hookworm infection. Transactions of the Royal Society of Tropical Medicine and Hygiene.

[B006] Chapman P. R., Giacomin P., Loukas A., McCarthy J. S. (2021). Experimental human hookworm infection: A narrative historical review. PLoS Neglected Tropical Diseases.

[B007] Constantinoiu C. C., Goullet M. S., Constantinoiu E. C., Scott J. L. (2015). Mucosal tolerance of the hookworm *Ancylostoma caninum* in the gut of naturally infected wild dogs. Parasite Immunology.

[B008] Dantas-Torres F., Otranto D. (2014). Dogs, cats, parasites, and humans in Brazil: opening the black box. Parasites & Vectors.

[B009] Dias S. R. C., Cunha D. E. S., Silva S. M., Santos H. A., Fujiwara R. T., Rabelo É. M. L. (2013). Evaluation of parasitological and immunological aspects of acute infection by *Ancylostoma caninum* and *Ancylostoma braziliense* in mixed-breed dogs. Parasitology Research.

[B010] Dracz R. M., Mozzer L. R., Fujiwara R. T., Lima W. S. (2014). Parasitological and hematological aspects of co-infection with *Angiostrongylus vasorum* and *Ancylostoma caninum* in dogs. Veterinary Parasitology.

[B011] Faust E. C., D’Antoni J. S., Odom V., Miller M. J., Peres C., Sawitz W., Thomen L. F., Tobie J., Walker H. (1938). A critical study of clinical laboratory technics for the diagnosis of protozoan cysts and helminth eggs in feces. American Journal of Tropical Medicine.

[B012] Feitosa F. L. F. (2014). Semiologia veterinária: A arte do diagnóstico.

[B013] Furtado L. F. V., Dias L. T. O., Rodrigues T. O., Silva V. J., Oliveira V. N. G. M., Rabelo É. M. L. (2020). Egg genotyping reveals the possibility of patent *Ancylostoma caninum* infection in human intestine. Scientific Reports.

[B014] Gibbs H. C. (1986). Hypobiosis in parasitic nematodes: An update. Advances in Parasitology.

[B015] Jain N. C. (1993). Essential of veterinary hematology..

[B016] Kaneko J. J., Harvey J. W., Bruss M. L. (2008). Clinical biochemistry of domestic animals.

[B017] Katagiri S., Oliveira‐Sequeira T. C. G. (2008). Prevalence of dog intestinal parasites and risk perception of zoonotic infection by dog owners in Sao Paulo State, Brazil. Zoonoses and Public Health.

[B018] Kopp S. R., Kotze A. C., McCarthy J. S., Coleman G. T. (2007). High-level pyrantel resistance in the hookworm *Ancylostoma caninum.*. Veterinary Parasitology.

[B019] Martinez C., Mooney C. T., Shiel R. E., Tang P. K., Mooney L., O’Neill E. J. (2019). Evaluation of red blood cell distribution width in dogs with various illnesses. The Canadian Veterinary Journal. La Revue Veterinaire Canadienne.

[B020] Meyer D. J., Harvey J., Meyer D. J., Harvey J. (2004). Veterinary Laboratory Medicine: Interpretation and Diagnosis.

[B021] Miglio A., Valente C., Guglielmini C. (2023). Red blood cell distribution width as a novel parameter in canine disorders: Literature review and future prospective. Animals (Basel).

[B022] Nagamori Y., Payton M. E., Looper E., Apple H., Johnson E. M. (2020). Retrospective survey of endoparasitism identified in feces of client-owned dogs in North America from 2007 through 2018. Veterinary Parasitology.

[B023] Neves D. P. (2004). Parasitologia Humana.

[B024] Oliveira-Arbex A. P., David E. B., Oliveira-Sequeira T. C. G., Katagiri S., Coradi S. T., Guimarães S. (2017). Molecular identification of *Ancylostoma* species from dogs and an assessment of zoonotic risk in low-income households, São Paulo State, Brazil. Journal of Helminthology.

[B025] Radakovich L. B., Pannone S. C., Truelove M. P., Olver C. S., Santangelo K. S. (2017). Hematology and biochemistry of aging-evidence of “anemia of the elderly” in old dogs. Veterinary Clinical Pathology.

[B026] Saldanha-Elias A. M., Silva M. A., Silva V. O., Amorim S. L. A., Coutinho A. R., Santos H. A., Giunchetti R. C., Vitor R. W. A., Geiger S. M. (2019). Prevalence of endoparasites in urban stray dogs from Brazil diagnosed with *Leishmania*, with potential for human zoonoses. Acta Parasitologica.

[B027] Santos B., Silva A. N. F., Mora S. E. V., Kozlowski V. A., Justo A. A., Pantoja J. C. F., Schmidt S. E. M., Takahira R. K. (2020). Epidemiological aspects of *Ancylostoma* spp. infection in naturally infected dogs from São Paulo state, Brazil. Veterinary Parasitology. Regional Studies and Reports.

[B028] Schmidt E. M. S., Tvarijonaviciute A., Martinez-Subiela S., Cerón J. J., Eckersall P. D. (2016). Changes in biochemical analytes in female dogs with subclinical *Ancylostoma* spp. infection. BMC Veterinary Research.

[B029] Stockham S. L., Scott M. A. (2011). Fundamentos da Patologia Clínica Veterinária.

[B030] Traversa D., Regalbono A. F., Cesare A., Torre F., Drake J., Pietrobelli M. (2014). Environmental contamination by canine geohelminths. Parasites & Vectors.

[B031] Wang C. H., Lee S. C., Huang S. S., Chang L. C. (2011). Hookworm infection in a healthy adult that manifested as severe eosinphilia and diarrhea. Journal of Microbiology, Immunology, and Infection.

[B032] Willis H. H. (1921). A simple levitation method for the detection of hookworm ova. The Medical Journal of Australia.

